# Risk Factors Associated With COVID-19 Symptoms and Potential Vertical Transmission During Pregnancy: A Retrospective Cohort Study

**DOI:** 10.7759/cureus.22900

**Published:** 2022-03-06

**Authors:** Bibita Peter, NIcholas Ree, Karen Ferrer, Laila Younes, Barbara Lepe, Khilfeh Manhal, Janardhan Mydam

**Affiliations:** 1 Neonatology, John H. Stroger, Jr. Hospital of Cook County, Chicago, USA; 2 Pathology, John H. Stroger, Jr. Hospital of Cook County, Chicago, USA; 3 Family Medicine, AMITA Health Saints Mary and Elizabeth Hospital, Chicago, USA; 4 Maternal Child Services, AMITA Health Saints Mary and Elizabeth Hospital, Chicago, USA

**Keywords:** sars-cov-2, pregnancy, hypokalemic, potassium, overweight, obesity, bmi, covid-19

## Abstract

Objective

The COVID-19 pandemic is of special concern for pregnant women. A growing body of evidence suggests the virus can have a deleterious impact upon outcomes related to birth and newborn health. There is a paucity of published research demonstrating the factors that influence disease severity among those who are pregnant, while a growing body of evidence demonstrates that vertical transmission occurs. Our study investigated the impact of maternal characteristics upon COVID-19 outcomes, as well as whether disease severity impacted pregnancy outcomes.

Methods

We conducted a retrospective cohort study of pregnant women with COVID-19 who were admitted to two public hospitals in our state between April-August, 2020. Pregnancy outcomes and clinical, laboratory, and placental data were collected.

Results

Thirty-four pregnant women tested positive for SARS-CoV-2. Among them, 55% (19/34) were symptomatic. Of those who were symptomatic, 68% (13/19) presented with fever and cough. Those with symptoms had a statistically significant higher pregestational mean body mass index (BMI) compared with asymptomatic women (35.7±7.9 vs 26.7±6.9, *P*=0.004). Screening of biochemical records demonstrated that symptomatic women had lower potassium levels compared with those who were asymptomatic (median: 3.70 mEq/L vs 4.30 mEq/L, *P*=0.009). The lowest potassium level (3.0 mEq/L) and one of the highest BMIs (42.4 kg/m^2^) was observed in the only case of postpartum mortality among the symptomatic women. We did not observe any influence of maternal COVID-19 severity on placental histopathology/infant health or evidence of vertical transmission.

Conclusion

High pregestational BMI and lower potassium levels were associated with the presence of COVID-19 symptoms among pregnant women.

## Introduction

As the global impact of coronavirus disease 2019 (COVID-19) has grown, so has our understanding of the plethora of presenting symptoms. Known symptoms now include anosmia, myalgia, and gastrointestinal symptoms, including diarrhea, vomiting, nausea, and abdominal pain, in addition to respiratory symptoms of fever, dry cough, and dyspnea [1-3]. It is also established that older age, underlying medical conditions (immunosuppression, hypertension, and diabetes), and race/ethnicity (Black, Hispanic and Asian) increase the risk of severe disease [4-6]. However, the impact of COVID-19 and associated risk factors on pregnancy and newborn health remains less certain.

A number of studies, including a large systematic review, suggest that the overall risk of vertical transmission and clinical symptoms in newborns of women with COVID-19 is small [1, 7, 8], although risks of neonatal complications are higher compared to infants of non-infected mothers [9]. However, evidence of the considerable effects of COVID-19 on maternal morbidity and mortality continues to accumulate [10,11]. A multinational study showed an increased risk of pre-eclampsia, severe infections, intensive care unit admission, preterm birth, and maternal mortality in pregnant women with COVID-19, relative to their uninfected counterparts [9]. With the recent implementation of aggressive COVID-19 testing, several reports have emerged associating factors such as pregestational body mass index (BMI), lymphocyte count, heart rate, and respiratory rate with the development of disease symptoms in SARS CoV-2-infected pregnant women [12-16]. Another study demonstrated an increased risk of preterm delivery in symptomatic women, suggesting a need to identify high-risk pregnant women [10]. One of the potential reasons for the increased risk could be damage to placental tissue [16].

Our study sought to investigate the impact of maternal demographic, clinical and biochemical characteristics on disease severity in pregnant women infected with SARS-Cov-2. The ability to identify factors that place pregnant women at particular risk of developing COVID-19 symptoms may help healthcare providers determine which women need to be closely monitored for a potential rapid progression of the illness. We further investigated the influence of disease severity on pregnancy outcomes, vertical transmission of infection, histopathological evaluation of the placenta, and outcomes of the newborn.

This article appeared as a preprint on Research Square on October 1, 2021. It was also presented as an electronic poster at the 2021 virtual meeting of the Pediatric Academic Societies (PAS 2021).

## Materials and methods

Study design and study population

During the peak of the first wave of COVID-19 infections in the US, testing for COVID-19 became universal for all pregnancy admissions from April 2020 in Illinois, USA. We conducted a retrospective cohort study between April 1 and August 15, 2020, that included all pregnant women who were admitted at any gestational age, at any stage of labor, and had laboratory-confirmed COVID-19. Admissions were from two Chicago maternity hospitals - John H. Stroger, Jr. Hospital of Cook County and AMITA Health Saints Mary and Elizabeth Medical Center. Our study was possible because of the April 2020 implementation of universal COVID-19 testing for all pregnant women admitted to hospitals in Illinois. We followed the World Health Organization (WHO) guidelines for diagnosis, which define positive real-time reverse transcriptase-polymerase chain reaction (RT-PCR) assay of nasal or pharyngeal swabs as laboratory-confirmed SARS-CoV-2 [17]. We excluded patients lacking the data on COVID-19 related symptoms (fever, cough, myalgia, anosmia, congestion, headache, chills, dyspnea, nausea, vomiting, or malaise) that would allow us to classify them as symptomatic or asymptomatic. We also excluded one woman with missing data on pregnancy-related complications, such as pregnancy-induced hypertension, chronic hypertension, pregnancy-related diabetes, or chronic diabetes (she did not have prenatal care). After the selection of the study population, we further included their newborns in the study.

Ethical approval and data collection

The study's protocol was expeditiously approved by the institutional research ethics committees associated with John H. Stroger, Jr. Hospital of Cook County (approval number: 20-098) and AMITA Health Saints Mary and Elizabeth Medical Center (approval number: 2021-0193-02). The requirement of informed consent was waived due to the retrospective study design.

The following demographic and baseline maternal data were collected: age, race and ethnicity, BMI, lifestyle habits (substance abuse, alcohol consumption, and smoking), comorbidities including pre-gestational obesity, hypertension, gestational diabetes, and whether the patient received antepartum therapy including hydroxychloroquine treatment. All participants underwent clinical evaluation of presenting signs and symptoms, detailed laboratory assessment of blood and urine samples, and radiologic chest assessment if needed.

Maternal blood sample assessment included hemoglobin, blood cell counts, inflammatory markers [e.g., C-reactive protein (CRP)], serum concentration of electrolytes (sodium, potassium, calcium, and chloride), liver function (alanine aminotransferase [ALT], aspartate aminotransferase [AST]), and renal function (blood urea nitrogen [BUN] and creatinine). Data on pregnancy outcomes (including mode of delivery, gestational age (GA), and preeclampsia), and neonatal outcomes [including symptoms, Appearance, Pulse, Grimace, Activity, and Respiration (APGAR) scores, and birth weight] were recorded. Preterm birth or premature birth was defined as one occurring at < 37 weeks. We used specific cut-off values to define blood dyscrasias for pregnant women according to their trimester: leukopenia as white cell count < 5.9 x 109/L, neutropenia as neutrophil count < 3.9 x 109/L, and lymphopenia as lymphocyte count < 1.0 x 109/L [18] for the third trimester. We also performed the gross and histopathological evaluation of placentas according to the Amsterdam consensus statement guidelines [19].

We further performed laboratory assessment of blood samples taken from the newborns and followed up both mother and infant until six weeks after delivery. We defined the infant's specific blood dyscrasias according to their age of life: leukopenia as white cell count < 13.0 x 109/L for term infants at one to 12 hours of life and < 9.0 x 109/L for preterm infants at birth; neutropenia as neutrophil count < 6.0 x 109/L for term infants at one to 12 hours of life and < 6.0 x 109/L for preterm infants at birth; and lymphopenia as lymphocyte count < 2.0 x 109/L for both term infants at one to 12 hours of life and preterm infants at birth [20]. Evidence of vertical transmission was further evaluated for the presence of SARS-CoV-2 according to CDC guidelines [21]. The guidelines require two PCR tests, the first at 24 hours and the second at 48 hours after birth, along with clinical monitoring for signs and symptoms of COVID-19. Our protocol for prevention of perinatal infection initially included separation of newborns from mothers (who were SARS-CoV-2 positive) after delivery, but later CDC guidelines left the decision to separate up to the mothers. In our participating hospitals, mothers who chose to care for their babies were instructed to practice strict handwashing and masking and to maintain social distancing (6 feet) when not caring for the baby, for either 14 days or four days following resolution of symptoms. As, and when, appropriate we evaluated infants for immunoglobulin [Ig] G and IgM levels.

All the data collected was curated using a customized data collection form, and two study investigators (JM and BP) independently reviewed the data collection forms for any errors. The data was locked and secured appropriately according to rules and principles laid down in the Health Insurance Portability and Accountability Act of 1996 (HIPAA). The data collected from both sites were synchronized and any inaccuracies were verified with the concerned representative of the specific center.

Study outcome

The patients were further classified into two groups: symptomatic and asymptomatic, according to the existence of any of the following known signs and symptoms of COVID-19 infection: fever, cough, myalgia, anosmia, congestion, headache, chills, desaturation, dyspnea, nausea, vomiting, or malaise. The symptomatic group included women who displayed symptoms at any time before or after delivery.

Statistical analyses

Statistical analyses were conducted using STATA 16 (StataCorp. 2019. Stata Statistical Software: Release 16. College Station, TX: StataCorp LLC) and SAS 9.4 (SAS Institute, Inc., Cary, NC). Continuous variables were expressed as mean and standard deviation (SD) for normally distributed data, median and range for non-normally distributed data, and categorical variables as frequency and percentage. The Shapiro-Wilk, Kolmogorov-Smirnov, Cramer-von Mise, and Anderson-Darling tests were used to check the normality of the data. The continuous variables were compared between symptomatic and asymptomatic individuals using a t-test or Mann-Whitney-U test and categorical variables were compared using the Pearson chi-square test or Fisher's-exact test, as appropriate. We calculated the unadjusted odds ratio (OR) using the simple logistic regression model. A p-value of < 0.05 was considered statistically significant.

## Results

**Figure 1:**
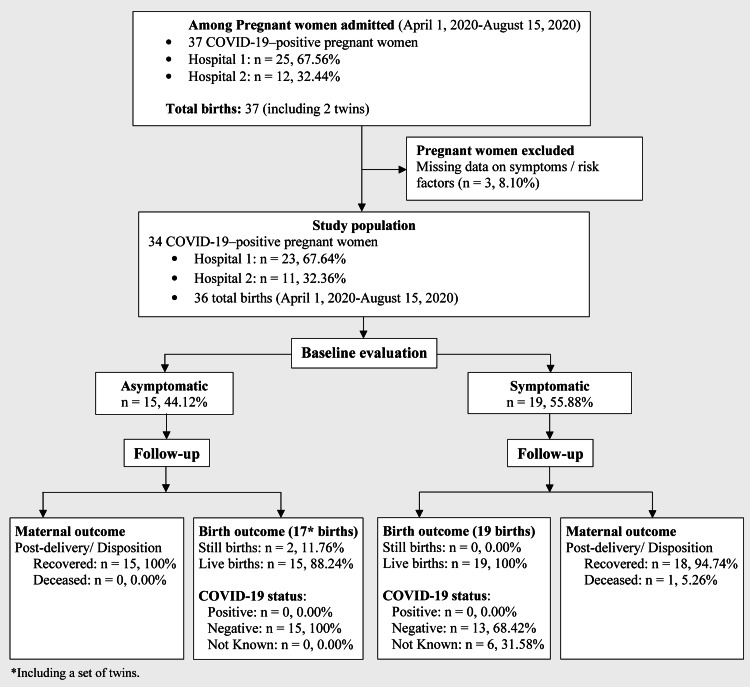
Flowchart of the study population selection and maternal and infant outcomes. Hospital 1 is John H. Stroger, Jr. Hospital of Cook County; Hospital 2 is AMITA Health Saints Mary and Elizabeth Medical Center.

A total of 37 pregnant women with COVID-19 were admitted to the study centers during the study period. One patient was excluded for lack of information on the existence of symptoms, while two were excluded due to lack of data related to maternal and perinatal outcomes. In total, 34 women were included in the study, and 19 were symptomatic (55%) for COVID-19 (Figure 1).

Baseline characteristics of pregnant women

**Figure 2:**
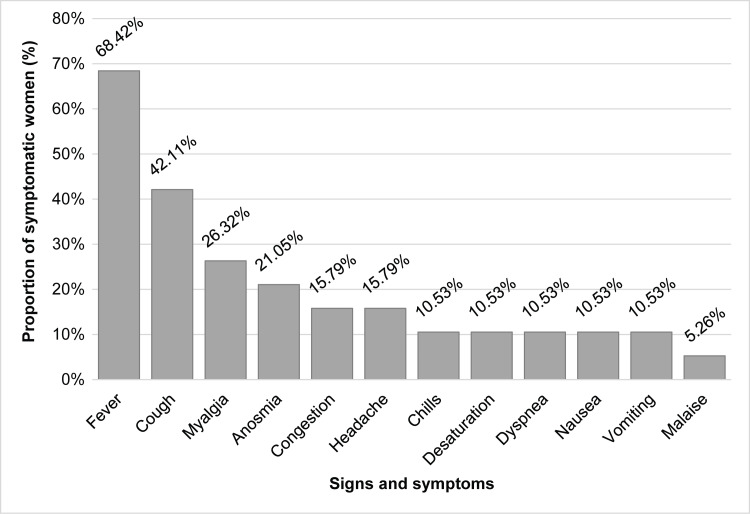
Distribution of symptoms, secondary to COVID-19 infection, among symptomatic pregnant women.

The baseline characteristics of the study population are shown in Table 1. Among the 19 symptomatic patients, fever (n=13; 68%), cough (n=8; 42%), and myalgia (n=5; 26%) were the most commonly observed symptoms (Figure 2). Fever, cough, and myalgia were the most frequently observed combination of coexisting symptoms, seen in three patients (15.79%; data not shown). The average age of women in our study population was 26. The largest proportion was of Hispanic ancestry (44%), followed by non-Hispanic Black (38%). The distribution of age and race and ethnicity did not differ significantly between symptomatic and asymptomatic patients.

**Table 1:** Clinical and demographic characteristics of pregnant women with COVID-19 by symptom status BMI: body mass index; GA: gestational age; GBS: Group B Streptococcus; HIV: human immunodeficiency virus; HSV: herpes simplex virus; HTN: hypertension; NA: not available; SD: standard deviation, IQR: Interquartile range. The Shapiro-Wilk, Kolmogorov-Smirnov, Cramer-von Mises, and Anderson-Darling tests were used to check the normality of the data. ^a^ Mean (SD), ^b^ Median (IQR), ^c^ Percentage (%)

Characteristics	Asymptomatic (N=15)			Symptomatic (N=19)		P
	n	Mean (SD)/ Median (IQR)/ %		n	Mean (SD)/ Median (IQR)/ %	
Age at diagnosis ^a^	15	24.93 (5.09)		19	27.32 (5.96)	0.227
Gravidity ^c^						
>1	9	60.00		15	78.95	0.276
Parity ^c^						
>1	5	33.33		8	42.11	0.601
GA at diagnosis (weeks) ^b^	15	39.00 (38.40-39.10)		18	35.90 (32.60-39.10)	0.173
GA at delivery (weeks) ^b^	14	39.05 (38.50-40.30)		19	39.10 (37.50-40.00)	1.000
Diagnosis to delivery interval (weeks) ^b^	14	0.05 (0-0.80)		19	2.10 (0-7.50)	0.035
Ethnicity ^c^						
Hispanic	7	46.67		8	42.11	1.000
Non-Hispanic black	6	40.00		7	36.84	
Non-Hispanic white	0	0.00		1	5.26	
Other	2	13.33		3	15.79	
Complications during pregnancy						
Pre-pregnancy BMI (kg/m2) ^a^	14	26.79 (6.92)		14	35.71 (7.91)	0.004
Obesity (BMI>30) ^c^	0	0.00		5	26.32	0.032
GBS positive ^c^	4	26.67		7	36.84	0.715
HIV positive ^c^	0	0.0		2	10.53	0.492
Preeclampsia ^c^	2	13.33		2	10.53	1.000
HTN ^c^	1	6.67		5	26.32	0.196
Cholestasis ^c^	0	0.00		2	10.53	0.492
Chorioamnionitis ^c^	0	0.00		3	15.79	0.244
Anemia ^c^	0	0.00		1	5.26	1.000
Gestational diabetes ^c^	0	0.00		2	10.53	0.492
Substance abuse ^c^	1	6.67		2	10.53	1.000
Depression ^c^	0	0.00		1	5.26	1.000
History of syphilis ^c^	1	6.67		0	0.00	0.441
History of HSV ^c^	0	0.00		1	5.26	1.000
Oligohydramnios ^c^	0	0.00		1	5.26	1.000
Relevant Antepartum therapy ^c^						
Flu vaccination in pregnancy	3	20.00		9	47.37	0.097
History of Malaria medication	1	6.25		0	0.00	NA
Antiviral	0	0.00		0	0.00	NA
Hydroxychloroquine	0	0.00		1	5.26	NA
Betamethasone	2	13.33		0	0.00	0.187
Labor and delivery						
Mode of delivery ^c^						
Vaginal delivery	11	73.33		14	73.68	1.000
Cesarean delivery	4	26.67		5	26.32	
Multiplicity of birth ^c^						
Twins (n, %)	2	13.33		0	0.00	0.187
Disposition/ Recovery ^c^						
Postpartum death, n (%)	0	0.00		1	5.26	1.000

**Figure 3:**
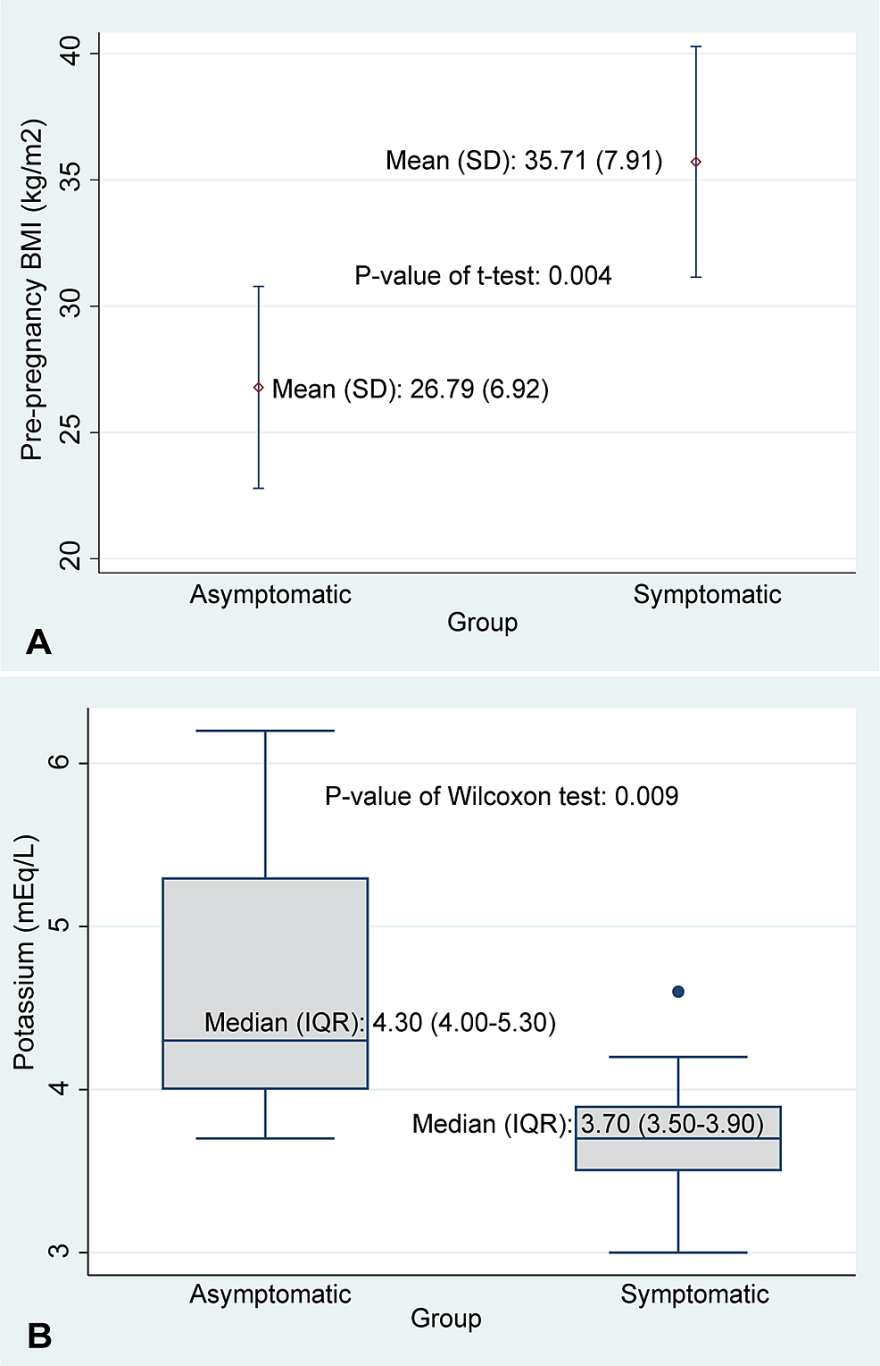
Distribution of pregestational BMI and potassium levels among pregnant women with COVID-19. A. Distribution of pregestational BMI by symptom status; B. Distribution of potassium levels by symptom status.

Obesity (n=5; 26.32%), gestational diabetes (n=2; 10.53%), and hypertension (n=5; 26.32%) were the most commonly occurring comorbid conditions. All three were overrepresented in symptomatic patients. We also observed that the symptomatic women had significantly higher pre-gestational BMI compared with asymptomatic women (35.71 vs 26.79, P=0.004; Figure 3A). Our posthoc power analysis for BMI with our sample size (n=34), mean difference of 8.92, standard deviations of 6.92 (group 1) and 7.91 (group 2), and an alpha error < 0.05 showed 92% as the power of the study.

Two of our symptomatic patients were also positive for HIV. Another three symptomatic women were diagnosed with chorioamnionitis. A comparatively large proportion of symptomatic women in our cohort had received the flu vaccination in the recent past (47.37% of symptomatic women vs 20.00% of asymptomatic women). We further observed that every third patient in our enrolled population was a Group B streptococcus (GBS) carrier; however, we failed to detect any influence of GBS status on the absence or presence of symptoms. Our cohort also had two women with a history of substance abuse, one symptomatic and one asymptomatic. We also detected syphilis in one of the asymptomatic patients.

We failed to detect any significant difference in the method or mode of delivery in symptomatic vs asymptomatic women (P=0.493). Of the four cesarean deliveries performed in asymptomatic women, two were for twins in a breech presentation and two were indicated by non-reassuring fetal status.

Laboratory characteristics of pregnant women

Initial evaluation of laboratory characteristics of the study population is shown in Table 2. Hematological analyses suggested significantly elevated basophil counts in symptomatic, compared with asymptomatic, women (P=0.035). However, we failed to observe any significant difference in blood dyscrasias, including leukopenia, neutropenia, and lymphopenia. No differences in kidney or liver function were observed. Symptomatic women had significantly lower potassium levels compared to asymptomatic women (median: 3.70 mEq/L [IQR: 3.50-3.90] vs. median 4.30 mEq/L [IQR: 4.00-5.30]; P=0.009; Figure 3B). The effect of lower potassium levels on disease severity was further observed to have a more profound effect in the presence of high BMI. We observed both low potassium levels (3.0 mEq/L, the lowest in the study) and high BMI (42.4 kg/m2, one of the highest in the cohort), in the only case of postpartum mortality among the symptomatic women in our study population. Three women, all of them symptomatic, showed evidence of secondary infection, with positive cell (n=1) and urine (n=2) cultures.

**Table 2:** Laboratory characteristics of pregnant women with COVID-19 by symptom status * CCHHS normal values, **AMITA Saint Mary’s normal values ALP: alkaline phosphate; ALT: alanine aminotransferase; aPTT: activated partial thromboplastin time; AST: aspartate aminotransferase; BUN: blood urea nitrogen; Ca: calcium; Hb: hemoglobin; Hct: Hematocrit; INR: international normalized ratio; K: potassium; Na: sodium; NA: not available; PT: prothrombin time; SD: standard deviation ^a^ Percentage (%), ^b^ Median (IQR), ^c^ Leucocyte count < 5.9 x 10^9^ cells/L, ^d^ Lymphocyte count < 10^9^ cells/L, ^e^ Neutrophil count <3.9 x 10^9^ cells/L.

Characteristics	Normal range	Asymptomatic (N=15)		Symptomatic (N=19)		P
		n	Median (IQR)/ %	n	Median (IQR)/ %	
Hematological profile						
Blood group ^a^						
A	NA	4	26.67	5	26.32	0.928
AB	NA	1	6.67	0	0.00	
B	NA	1	6.67	2	10.53	
O	NA	9	60.00	12	63.16	
Rhesus status ^a^						
Negative	NA	0	0.00	2	10.53	0.492
Positive	NA	15	100	17	89.47	
Hct (%)^b^	34.9-44.3*/34.7-45.1**	15	34.00 (30.80-36.30)	17	33.90 (30.80-36.40)	0.940
Leukocyte count (K/uL) ^b^	4.4-10.6*/4.0-11.0**	15	8.80 (7.30-11.30)	16	9.70 (5.70-10.60)	0.621
Leukopenia^c^						
Yes ^a^	NA	2	13.33	5	31.25	0.394
Lymphocyte count (K/uL) ^b^	1.2-3.4*/0.6-3.4**	10	16.75 (11.00-21.60)	9	15.40 (11.00-24.60)	0.902
Lymphopenia^d^						
Yes ^a^	NA	0	0.00	0	0.00	NA
Basophil count (K/uL) ^b^	0-0.1*/0-0.2**	10	0.05 (0-0.20)	9	0.30 (0.30-0.60)	0.035
Neutrophil count (K/uL) ^b^	2.2-6.9* 1.7-7.7**	10	72.70 (68.10-76.00)	7	72.80 (65.90-77.20)	1.000
Neutropenia^e^
Yes ^a^	NA	0	0.00	0	0.00	NA
Platelet count (K/uL) ^b^	161-369*/150-450**	14	216.00 (157.00-239.00)	16	209.50 (156.50-239.50)	0.950
Blood biochemistry profile						
ALT (U/L) ^b^	5‐35*/0-50**	5	12.00 (8.00-20.00)	13	12.00 (10.00-21.00)	0.519
AST (U/L) ^b^	0‐40*/0-40**	5	20.00 (20.00-23.00)	13	17.00 (15.00-26.00)	0.236
Glucose mg/dL ^b^	65-110*/70-99**	7	93.00 (69.00-102.00)	14	88.50 (74.00-119.00)	1.000
Albumin (g/dL) ^b^	3.8-5.2*/3.5-5.7**	4	2.95 (2.80-3.20)	12	3.10 (2.90-3.20)	0.760
Total bilirubin (mg/dL) ^b^	0.2-1.2*/0.0-1.0**	4	0.60 (0.450-1.15)	12	0.60 (0.35-0.90)	0.668
ALP (U/L) ^b^	20‐120*/35-104**	4	198.00 (155.00-284.50)	12	147.00 (95.50-183.50)	0.163
Creatinine (mg/dL) ^b^	0.6-1.4*/0.5-1.2**	5	0.60 (0.50-0.80)	12	0.53 (0.50-0.63)	0.387
Na (mEq/L) ^b^	135‐145*/133-144**	6	135.50 (134.00-137.00)	13	136.00 (133.00-138.00)	0.894
K (mEq/L) ^b^	3.5‐5.0*/3.5-5.1**	7	4.30 (4.00-5.30)	13	3.70 (3.50-3.90)	0.009
Ca (mg/dL) ^b^	8.5-10.5*/8.6-10.3**	4	8.35 (8.00-8.65)	12	8.20 (8.10-8.35)	0.903
BUN (mg/dL) ^b^	8-20*/7-25**	5	5.00 (4.00-10.00)	12	5.00 (4.00-7.00)	0.631
Protein (g/dL) ^b^	6.4-8.3*/6.4-8.9**	4	5.50 (5.05-6.35)	12	5.75 (5.60-6.00)	0.670
Coagulation profile						
PT (s) ^b^	11.9-14.1*	7	12.70 (11.90-14.60)	7	13.10 (11.60-13.40)	0.848
aPTT (s) ^b^	25.1‐36.5*	7	31.70 (26.20-33.50)	6	30.45 (30.10-32.00)	0.943
INR ^b^	0.9-1.1**	7	1.00 (0.95-1.17)	7	1.01 (0.77-1.04)	0.654
Fibrinogen (mg/dL) ^b^	178-454*/163-463**	7	419.00 (364.00-533.00)	6	456.00 (361.00-533.00)	1.000
Microbiological profile						
Blood culture ^a^						
Negative		2	100.00	4	80.00	1.000
Positive		0	0.00	1	20.00	
Urine culture ^a^						
Negative		2	100	3	60.00	1.000
Positive		0	0.00	2	4.00	

Placental histopathological features

**Figure 4:**
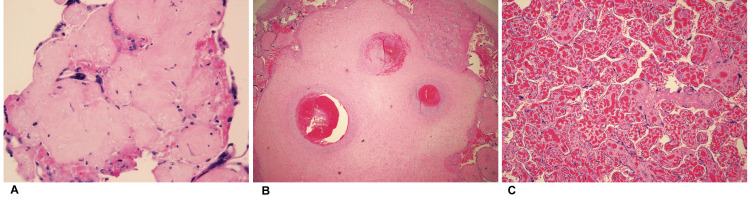
Placental pathology unique to symptomatic women with COVID-19 in our study population. A. Fetal vascular malperfusion (FVM), avascular villi type (n=1); B. FVM, hemorrhagic endovasculitis type (n=1); C. Chorangiosis (n=4).

We found no association of specific histopathological placental features with signs and symptoms of COVID-19 (Table 3). However, we did observe a higher prevalence of fetal vascular malperfusion (FVM), immunological or inflammatory processes, and chorangiosis in symptomatic women compared to asymptomatic women. FVM (n=2; one avascular villi type and one hemorrhagic endovasculitis type) and chorangiosis (n=4) were observed exclusively in the placentas of symptomatic patients (Figure 4).

**Table 3:** Placental pathology of pregnant women with COVID-19 by symptom status IM: inflammatory or immune process; FVM: fetal vascular malperfusion; MVM: maternal vascular malperfusion; SD: standard deviation. ^a^ Percentage (%), ^b^ Mean (SD), *Avascular villi type (n=1), hemorrhagic endovasculitis type (n=1).

Characteristics		Asymptomatic (N=15)			Symptomatic (N=19)	P
	n	Mean (SD)/ %		n	Mean (SD)/ %	
MVM ^a^						
No	3	25.00		6	31.58	1.000
Yes	9	75.00		13	68.42	
FVM ^a^						
No	12	100		17	89.47	0.510
Yes	0	0.00		2*	10.53	
IM processes ^a^						
No	7	58.33		7	36.84	0.242
Yes	5	41.67		12	63.16	
Chorangiosis ^a^						
No	12	100		15	78.95	0.139
Yes	0	0.00		4	21.05	
Intervillous thrombus ^a^						
No	9	75.00		17	89.47	0.350
Yes	3	25.00		2	10.53	
Placental weight ^b^	11	489.00 (122.87)		16	506.13 (96.44)	0.689

Logistic regression analysis of significant maternal variables

Simple logistic regression analysis showed that the odds of severe COVID-19 increased as pre-gestational BMI increased and pre-delivery potassium levels decreased (Table 4). For each unit increase in pre-gestational BMI, the odds of being symptomatic due to SARS Cov-2 infection increased by 18% [odds ratio (OR): 1.18; 95% confidence interval (CI): 1.03 - 1.34)], while a one-unit decrease in serum potassium levels among pregnant women before delivery increased the odds of symptomatic infection by 19.72 (OR: 19.72; 95% CI: 1.03 - 376.79).

**Table 4:** Simple logistic regression model of predictors of symptomatic COVID-19 in pregnant women. *In the calculation of the odds ratio for potassium, negative one unit (i.e., unit = −1) was used to calculate the increase in the odds of symptomatic infection per one unit decrease in potassium level. CI: Confidence interval.

Variable	Odds ratio (OR)	95% CI
Pre-pregnancy BMI (kg/m2)	1.18	(1.03 - 1.34)
Potassium (mEq/L)*	19.72	(1.03-376.79)

Fetal and neonatal outcomes

Among 34 deliveries, there were two twin deliveries culminating in a total of 36 infants in our study population (Table 5). Mean birth weight was 3332.50g (SD=778.37) with a median GA of 39.10 weeks (IQR: 38.00-40.00). The average birth weight of babies born to symptomatic women was significantly higher than that of asymptomatic women (mean: 3454.00g [SD: 521.11] vs 2885.80g [SD: 941.09], P=0.048). We observed three premature deliveries (GA< 37 weeks), with one resulting in twin fetal demise. In contrast to the mothers, the majority of newborns were asymptomatic, with tachypnea most likely secondary to transient tachypnea observed as the most common symptom in five symptomatic newborns. Among five newborns with tachypnea, four were born to symptomatic mothers. However, none of the newborns tested positive for COVID-19.

**Table 5:** Clinical and demographic characteristics of infants born to women with COVID-19, by maternal symptom status *Includes one pair of twins NA: not available; RDS: respiratory distress syndrome; RT-PCR: reverse transcriptase-polymerase chain reaction; TTN: transient tachypnea of newborn; SCN/NICU: Special Care Nursery/ Neonatal Intensive Care Unit; GERD: gastroesophageal reflux disease. ^a^ Mean (SD), ^b^ Median (IQR), ^c^ Percentage (%)

Characteristics		Asymptomatic (N=15)			Symptomatic (N=19)	P
	n	Mean (SD)/ Median (IQR)/ %		n	Mean (SD)/ Median (IQR)/ %	
Weight of newborns, g ^a^	15	2885.80 (941.09)		19	3454.00 (521.11)	0.048
Premature birth (<37 weeks) ^b^	3	21.43		0	0.00	0.067
Stillbirth* ^b^	2	13.33		0	0.00	
Sex ^b^						
Male	9	60.00		9	47.37	0.464
Female	6	40.00		10	52.63	
Apgar scores at 1 minute ^c^	15	9.00 (9.00-9.00)		19	9.00 (8.00-9.00)	0.790
Apgar scores at 5 minutes ^c^	15	9.00 (9.00-9.00)		19	9.00 (9.00-9.00)	0.724
RT PCR for SARS-Cov2 ^b^						
Positive	0	0.00		0	0.00	NA
Negative	15	100.00		13	68.42	
Not done	1	0.00		6	31.58	
Initial temperature	15	36.80 (36.50-37.10)		18	36.70 (36.50-37.00)	0.971
Symptoms ^b^						
Apnea	1	7.14		0	0.00	0.424
Increased work of breathing	2	14.29		3	15.79	1.000
Desaturations	2	14.29		4	21.05	0.618
Hypoglycemia	0	0.00		1	5.26	1.000
Poor feeding	0	0.00		2	10.53	0.496
Fever	1	7.14		0	0.00	0.424
Diagnosis ^b^						
Normal	9	60.00		9	50.00	0.566
RDS	2	13.33		1	5.26	0.571
Rule out sepsis	3	20.00		0	0.00	0.076
TTN	0	0.00		2	10.53	0.492
Apnea	1	6.67		0	0.00	0.441
Pneumomediastinum	0	0.00		1	5.26	1.000
Poor feeding/GERD	0	0.00		1	5.26	1.000
Hypoglycemia	0	0.00		1	5.26	1.000
Birth Injury	0	0.00		1	5.26	1.000
Disposition ^b^						
SCN/NICU visit and discharged	6	40.00		9	50.00	0.566
Nursery/Postpartum floor visit and discharged	9	60.00		9	50.00	

Laboratory characteristics of newborns

We did not detect any difference in laboratory characteristics in infants of symptomatic women compared with those of asymptomatic women (Table 6). We also measured total IgM (n=6) and total IgG (n=7) antibodies in a limited number of infants (Table 6). Our data suggest higher levels of IgM antibodies in one infant (17.00) born to a symptomatic mother compared with four infants born to asymptomatic mothers (median: 10.00, IQR: 9.00-11.00), but the low sample size makes it impossible to make any meaningful clinical interpretation.

**Table 6:** Laboratory characteristics of infants born to women with COVID-19, by maternal symptom status * CCHHS normal values, **AMITA Saint Marys normal values; ALT: alanine aminotransferase, AST: aspartate aminotransferase, BUN: blood urea nitrogen, CRP: C-reactive protein, Hct: Hematocrit, Ig: immunoglobulin, NA: not available. ^a^ Median (IQR), ^b^ Percentage (%), ^c^ Leucocyte count <9.0 x 10^9^ cells/L for preterm infants and 13.0 x 10^9^ cells/L for term infants, ^d^ Lymphocyte count <2.0 x 10^9^ cells/L, ^e^ Neutrophil count < 6.0 x 10^9^ cells/L.

Characteristics	Normal Range	Asymptomatic (N=15)		Symptomatic (N=19)		P
		n	Median (IQR)/ %	n	Median (IQR)/ %	
Hematological profile						
Hct (%) ^a^	42-60.0*/42.0-54.0**	6	46.40 (42.35-53.80)	14	50.90 (47.30-52.70)	0.322
Leucocyte count (K/uL) ^a^	9.1-34.0*/8.0-15.4**	8	13.00 (9.80-17.95)	14	13.85 (11.30-17.30)	0.657
Leukopenia^c^						
Yes ^b^	NA	2	28.57	5	35.71	1.000
Lymphocyte count (%) ^a^	11.0-30.9*/33.7-67.6**	7	26.00 (19.00-32.00)	14	22.00 (14.00-30.00)	0.501
Lymphopenia^d^						
Yes ^b^	NA	0	0.00	0	0.00	NA
Basophil count (%) ^a^	0.0-0.3*/0.1-0.8**	5	2.00 (0.00-5.00)	7	0.00 (0.00-1.00)	0.487
Monocyte count (%) ^a^	0.0-10.0*/6.7-19.9**	3	9.00 (8.00-12.00)	8	13.20 (8.50-15.90)	0.357
Neutrophil count (%) ^a^	65.9-69.1*/20.2-46.2**	7	50.00 (45.00-58.00)	14	60.50 (52.00-73.00)	0.192
Neutropenia^e^						
Yes ^b^	NA	0	0.00	0	0.00	NA
Platelet count (K/uL) ^a^	200-400*/145-262**	8	192.00 (179.00-221.50)	14	245.50 (150.00-290.00)	0.232
Eosinophils count (%) ^a^	0.3-5.2**	3	3.00 (4.00-4.00)	6	1.55 (3.00-3.00)	0.691
Blood biochemistry profile						
CRP (mg/ dL) ^a^	<1.0**	3	0.10 (0.07-2.09)	8	0.09 (0.04-0.21)	0.409
ALT (U/L) ^a^	5-35*/0.0-40.0**	3	10.00 (9.00-17.00)	6	13.50 (11.00-19.00)	0.519
AST (U/L) ^a^	0-40*/0-32**	2	42.50 (25.00-60.00)	5	49.00 (37.00-57.00)	0.847
Creatinine (mg/dL) ^a^	0.6-1.4*/0.5-1.2**	3	0.70 (0.70-0.80)	6	0.86 (0.60-1.00)	0.693
BUN (mg/dL) ^a^	8-20*/7-25**	3	9.00 (7.00-28.00)	5	8.00 (6.00-8.00)	0.368
Blood culture ^b^						
Negative		7	100	9	100	NA
Positive		0	0.00	0	0.00	
Immunological profile						
IgM (mg/dL) ^a^	3-13*/14-142 **	5	10.00 (9.00-11.00)	1	17.00 (17.00-17.00)	0.373
IgG (mg/dL) ^a^	74-1421*	5	724.00 (706.00-868.00)	2	560.00 (20.00-1100.00)	0.847

## Discussion

To the best of our knowledge, this is the largest cohort of mother-baby dyads (SARS-Cov-2-positive women and their newborn infants) to undergo detailed clinical and biochemical investigation in the state of Illinois. In this study, high BMI and low potassium levels were associated with symptomatic COVID-19 in mothers.

About half of pregnant women in our study developed symptoms of COVID-19, which falls in the middle range of similar studies. A recent study of 70 pregnant women with SARS-CoV-2 in New York City reported that only 21% presented with symptoms [22], while an earlier study from Wuhan, China showed 95% of pregnant women with the virus exhibited symptoms [23].

We found that women with a higher pre-gestational BMI were more likely to be symptomatic, consistent with several reports showing an association between pre-gestational BMI with severe maternal outcomes among women with COVID-19 [12,14]. An Italian study observed significantly higher pre-gestational BMI in seven of 14 women showing severe symptoms [12], and a case series from Washington State found that the majority of pregnant women with severe infection were overweight or obese [14].

Our finding of significantly lower potassium levels in symptomatic women is consistent with a report of lower potassium levels (< 3.5 mEq/L) in 119 patients (41%) of 290 with COVID-19 in the general population [24]. In that study, hypokalemic patients were more likely to stay longer in hospitals, with a higher rate of respiratory symptoms. SARS-CoV-2 enters cells by binding to angiotensin-converting enzyme 2 (ACE2), leading to ACE2 depletion in affected cells [25]. ACE2 depletion promotes vasoconstriction, increasing the reabsorption of water and sodium [26]. These changes could lead to increased potassium excretion in symptomatic patients who may have higher viral loads [27]. Also, the contribution of respiratory alkalosis or diarrhea to low potassium levels among pregnant women with COVID-19 cannot be ruled out. Since low potassium levels could lead to life-threatening conditions, including cardiac events, our findings suggest the need to monitor serum potassium and improve care for pregnant women with COVID-19 by ensuring adequate potassium supplementation.

Hispanic and non-Hispanic Black populations comprised 44% and 38% of the pregnant women in our study population, respectively. A recent study of 1,567 Hispanic pregnant and postpartum women identified obesity as a major risk factor for moderate and severe COVID-19 [28], consistent with our finding that BMI was associated with symptomatic infection. We did not observe an association between Hispanic race/ethnicity and symptomatic COVID-19, but ours was a small study, and a larger study encompassing a broader geographic area may have yielded different results.

Our results also showed significantly higher basophil cell count in symptomatic patients compared with asymptomatic patients. A recent study demonstrated an association of elevated basophil levels with the IgG antibodies against SARS-CoV-2 produced by B cells during the disease's recovery phase, suggesting the possibility of heightened humoral response in symptomatic pregnant women [29].

We did not find a statistically significant association of specific placental pathologies with COVID-19 symptom status among the women in our study, and this result is consistent with a recent report by Hecht et al [30]. However, we did observe a spectrum of histopathologies (chorangiosis and FVM ) exclusively among symptomatic women, which were similar to inflammatory changes observed in placentas from a case series of fetal demise associated with maternal SARS-Cov-2 infection [16].

Limitations

Our study is limited by the small sample size and the retrospective study design. The retrospective data collection contributed to missing values and an increased likelihood of information bias. The sample size was too small to perform a robust multivariable analysis, so we were unable to assess potential predictive variables or evaluate confounding. However, our posthoc power analysis showed that our sample size of 34 women achieved a power of 92%, which is higher than the 80% standard generally seen in the literature. Finally, because the participating hospitals draw from largely an urban population of Chicago, our findings may not be generalizable to communities in other areas of the United States.

Even so, our findings have important implications for the clinical management of pregnant women with COVID-19. Our study suggests that pregnant women who test positive for SARS-CoV-2 should be closely monitored for a rapid progression of symptoms, especially women with a high pre-gestational BMI and/or low potassium levels. However, it remains to be seen whether low potassium levels are a cause or outcome of symptoms observed in pregnant women with COVID-19.

## Conclusions

The implications of SARS-CoV-2 infection in pregnant women are still not fully understood. However, our study observed that some pregnant women may be especially vulnerable to symptomatic infection. Our case series found that women with a high pregestational BMI had a higher risk of developing symptomatic COVID-19. The lower potassium levels we observed in symptomatic women could be a function of disease severity, or alternatively, could be a causal factor for the development of symptoms. We are unable to discern causality because of the retrospective nature of the study. Nevertheless, our study validates previous reports of an association of potassium levels with disease severity. We thereby strongly recommend closely following potassium levels to monitor disease severity in pregnant women with COVID-19 infection. Potassium levels may also be useful as a biomarker of symptom development. Finally, although it is encouraging that none of the newborns in our study tested positive for the virus, further research on the risks of vertical transmission for infants of women with COVID-19 during pregnancy is needed.
